# Clinicopathologic characteristics and outcomes of patients with mycosis fungoides: A single tertiary center retrospective analysis in Saudi Arabia

**DOI:** 10.15537/smj.2023.44.4.20220860

**Published:** 2023-04

**Authors:** Faisal A. Alghubaywi, Sawsan A. Alharthi, Sarah S. Aldharman, Raneem H. Najjar, Majed Y. Aleissa, Ohoud Z. Aljarbou, Mohammed I. AlJasser, Mohammad A. Almohideb

**Affiliations:** *From the College of Medicine (Alghubaywi, Aldharman, Najjar, Aleissa, AlJasser, Almohideb), King Saud bin Abdulaziz University for Health Sciences; from King Abdullah International Medical Research Center (Aleissa, AlJasser, Almohideb); from the Division of Dermatology (Alharthi, Aleissa, AlJasser, Almohideb), and from the Division of Pathology (Aljarbou), King Abdulaziz Medical City, Ministry of National Guard Health Affairs, Riyadh, Kingdom of Saudi Arabia.*

**Keywords:** neoplasms, cutaneous T-cell lymphoma, mycosis fungoides, Saudi Arabia

## Abstract

**Objectives:**

To determine the distinctive clinical and pathological characteristics and outcomes of MF in a Saudi population.

**Methods:**

We conducted a retrospective analysis of all MF cases diagnosed clinically with pathological confirmation at King Abdulaziz Medical City in Riyadh from January 2016 to July 2022. Variables include demographical, clinical, and pathological MF traits and disease outcomes.

**Results:**

A sum of 73 patients were enrolled. The mean age was 44 years. The female-to-male ratio was 1.3:1.

The mean duration between cutaneous manifestations and MF diagnosis was 33 months (2.7 years). Classic MF was the most common variant (60.3%), followed by hypopigmented MF (20.5%). Most patients (82.2%) had early-stage MF (IA, IB, and IIA). Patients who had CD4+/CD8+ with CD8 predominance had a favorable disease course (*p*=0.029). Topical corticosteroids were the most frequently prescribed treatment (79.5%). Three patients (4.1%) died from MF. The disease-specific survival rate for advanced-stage MF was 84.6%, which was significantly lower compared to early-stage MF (*p*=0.032).

**Conclusion:**

Among the Saudi population, MF has an earlier onset and slightly higher prevalence in females. Hypopigmented MF is more prevalent in this ethnic group. Immunohistochemical staining of CD4+/CD8+ with CD8 predominance may elucidate a favorable disease course.

**P**rimary cutaneous T cell lymphoma (CTCL) refers to a diverse group of T cell neoplasms that manifest mainly in the skin without predilection of extracutaneous sites upon diagnosis.^1^ Mycosis fungoides (MF) is the most prevalent subtype of CTCL, amounting to about half of all primary cutaneous lymphomas.^1^ In Europe, it accounts for approximately 4% of all cases of non-Hodgkin lymphoma.^2^ It substantially affects adults between the age of 55–60 years and confers a predilection to males with a ratio of 2:1.^1^ Reported originally in 1806 by the French dermatologist Jean Louis Alibert, MF is characterized by erythematous, atrophic, scaly patches with or without plaques, mostly over sun-protected skin that can evolve in a step-wise progression to tumor nodules and erythroderma.^3^ Multiple clinical variants have emerged since then, while the original presentation remains the “classical form.” Other established morphological forms of MF include hypopigmented, erythrodermic, and poikilodermatous.^4^

The pathogenesis of MF is presumed to be the consequence of genetic and epigenetic abnormalities that lead to chronic antigenic stimulation resulting in uninhibited clonal proliferation and the buildup of T cell helper memory cells in the skin.^5^ Due to the genetic heterogenicity among ethnicities, varying clinical characteristics of MF are observed in different demographical populations.^8,12,14^

Histologically, MF typically presents with superficial lymphoid infiltrate and profound epidermotropism of cytologically atypical T lymphocytes.^6^ These findings resemble features of many inflammatory diseases, and a repeat biopsy is often warranted. Clinicopathologic correlation is the gold standard of diagnosis, while equivocal cases may require immunohistochemical staining and molecular studies.^7^ With respect to tumor, node, metastasis, and blood (TNMB) stage and the patient’s overall health status, different outcomes have been noted in various geographical populations.^8,9^

Most studies of large MF cohorts have been conducted in Asian or Western countries and concluded diversified results depending on ethnic backgrounds. Recently, very few studies have investigated MF in the Saudi population.^10,11^ This study aimed to assess the clinical features, pathology, and survival analysis of MF in this ethnic group.

## Methods

This single-center retrospective study was carried out at King Abdulaziz Medical City, Riyadh, Saudi Arabia. The study was validated by the Ethics Committee of King Abdullah International Medical Research Center (IRB/1589/22).

Patients with biopsy-proven MF from January 2016 to July 2022 were included. Data were acquired by searching electronic medical records. Study variables were as follows: i) demographical data such as age and gender; ii) clinical characteristics, including initial misdiagnosis, duration between symptoms onset and diagnosis, MF stage, cutaneous location and extent of the disease, and the clinical variant of the disease; iii) histopathological data, including immunohistochemistry and relevant molecular studies; iv) clinical outcomes, including treatment modality and response (complete remission, partial response, stable disease, progressive disease, or relapse after complete remission), duration of treatment, and survival.

### Statistical analysis

Data were analyzed using SPSS version 25 (IBMCorp, Armonk, NY, USA). Categorical variables were described as frequencies and percentages. Numerical variables were presented as means and standard deviations. Additionally, bivariate analysis was done using the Chi-squared test and Kaplan–Meier survival analysis, which was achieved by a log-rank test. Statistical significance was set at *p*<0.05.

## Results

Eighty-two patients were found in the initial screening. Nine patients were excluded due to doubtful clinical presentation or absent histological correlation. A total of 73 patients with confirmed MF were analyzed. Table 1 outlines the demographic and clinical traits of MF in all patients. Females comprised 57.5% of the population (n=42). The male-to-female ratio was 1:1.3. The mean age at diagnosis was 44±15 years. Seventeen (23.3%) patients were 30 years old or younger. A family history of lymphoma or skin cancer was found in 3 (4.1%) patients.

**Table 1:** - Patient demographics and clinical disease characteristics (N=73).

Characteristic	n	%
***Gender***		
Male	31	42.5
Female	42	57.5
Male-to-female ratio (1:1.3)		
***Age at diagnosis***:	44±15.474	
Less than 18	4	5.5
From 18 to 30	13	17.8
From 31 to 50	25	34.2
From 51 to 70	30	41.1
71 years and above	1	1.4
***Initial misdiagnosis***		
None	43	59
Atopic dermatitis	15	20.5
Psoriasis	3	4.1
Parapsoriasis	3	4.1
Pityriasis Lichenoides	5	6.8
Other*	4	5.5
Time from cutaneous manifestations to MF diagnosis (months)	3.1±1.3	
Clinical phase		
Patch	52	71.2
Plaque	31	42.5
Tumors	1	1.4
***Clinical variant***		
Classic	44	60.3
Hypopigmented	15	20.5
Poikilodermatous	7	9.6
Erythrodermic	6	8.2
Hyperpigmented	1	1.4
***Anatomic site***		
Generalized	17	23.3
Head and neck	4	5.5
Trunk	40	54.8
Buttocks	10	13.7
Upper extremities	39	53.4
Lower extremities	44	60.3
Hands and feet	5	6.8
Body surface area (%)	20.95±25.38	
***Early stage MF***		
IA	34	46.6
IB	23	31.5
IIA	3	4.1
***Advanced stage MF***		
IIB	1	1.4
III	9	12.3
IVA	0	0.0
IVB	3	4.1
***Other clinical findings***		
Pruritus	39	53.4
Alopecia	3	4.1
Papules	4	5.5
Erosions	6	8.2
Superinfections	1	1.4
Other^§^	2	2.7
None	32	43.8

MF: mycosis fungoides *One lichen planus, one post-inflammatory hyperpigmentation, one lichen simplex chronicus, and one discoid Eczema. §Two had B symptoms, and one of them had peeling of the skin as well.

Mycosis fungoides was initially clinically misdiagnosed as atopic dermatitis in 15 (20.5%), pityriasis lichenoides in 5 (6.8%), or psoriasis in 3 (4.1%) patients. The mean duration from the appearance of the first cutaneous manifestation to MF diagnosis was 33.1 months (2.75 years). Sixty (82.2%) patients had early-stage MF (stages IA, IB, and IIA). The most frequent clinical presentations were patches (71.2%) and plaques (42.5%). The prevalent clinical variant was classic MF (60.3%), followed by hypopigmented MF (20.5%). Among the patients, four were children (11, 13, 13, and 15 years), all with hypopigmented MF. The mean body surface area was 21%±25%. Lesions were pruritic in 39 subjects (53.4%).

Table 2 summarizes all studied elements of histopathology. Immunohistochemistry was performed on 36 patients. Most had positive CD4, while CD7 was positive in only 2 (5.71%). The majority had CD4+/CD8+ with CD4 predominance (72.7%). Among all 73 pathology reports, large cell transformation (LCT) was present in 4 (5.48%) patients and Sézary cells in 2 (2.74%). T cell receptor (TCR) gene rearrangement testing on skin tissue was carried out in 32 patients; 18 (56%) were clonal (such as positive). The clinical disease course and outcomes were not affected in patients with LCT (*p*=0.167). Patients with CD4+/CD8+ with CD8 predominance generally had a better clinical course and outcomes in comparison to CD4+/CD8+ with CD4 predominance (*p*=0.029) (Table 3).

**Table 2:** - Findings of histopathology, immunohistochemistry, and molecular studies.

Characteristic	CD3	CD4	CD7	CD8	All
Total positive (n)	29	36	2	33	100
Total tested (n)	30	36	35	36	137
Positivity (%)	96.67	100	5.71	91.67	
	CD4+/CD8+, predominantly CD4	CD4+/CD8+, predominantly CD8	Large cell transformation	Sezary cells	TCR gene rearrangement
Total positive (n)	24	9	4	2	18
Total tested (n)	33	33	73	73	32
Percentage (%)	72.7	27.3	5.5	2.7	56.3

**Table 3:** - Relation between CD4:CD8 ratio and disease outcomes.

Characteristic	Outcome				*P*-value
	Complete remission n (%)	Partial response n (%)	Stable disease n (%)	Progressive disease n (%)	
CD4:CD8 ratio					
1:2	2 (33.3)	1 (12.5)	1 (6.3)	1 (20.0)	0.029
1:3	0 (0.0)	1 (12.5)	3 (18.8)	0 (0.0)	
2:1	4 (66.7)	0 (0.0)	4 (25.0)	1 (20.0)	
3:1	0 (0.0)	6 (75.0)	8 (50.0)	3 (60.0)	

**Figure 1:**
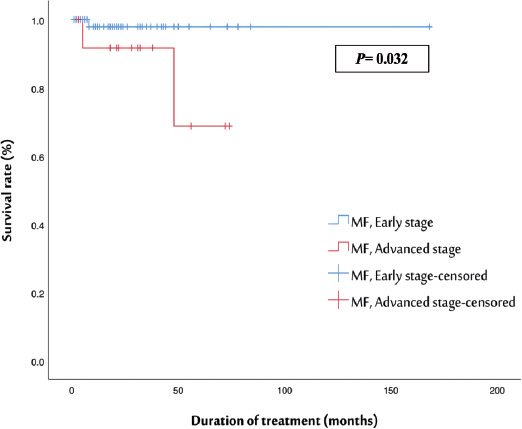
- Percentages of all treatment modalities used in our patients.

Figure 1 outlines all treatment modalities used in our patients. Topical corticosteroids were the most prevalent therapy across all MF stages (79.5%). Treatments and outcomes in each MF stage are shown in Table 4. Patients with early-stage MF mostly received skin-directed therapies, such as topical corticosteroids (67.1%) and narrow-band ultraviolet B (NBUVB) (51%). Patients with advanced-stage MF were treated using systemic modalities, such as chemotherapy (8.2%) combined with skin-directed therapy. The mean follow-up duration was 34.6 months. In the last appointment, 41.1% of the patients had stable MF; more than half (53.3%) of them were in stage IA. Eleven (15.1%) patients had complete remission, and 3 (4.1%) patients died secondary to MF.

**Table 4:** - Treatment modalities and outcomes by mycosis fungoides stage.

Characteristics	Stages						All
	IA	IB	IIA	IIB	III	IVB	
***Treatment modality***							
Topical corticosteroids	25 (43.1)	22 (37.9)	2 (3.4)	1 (1.7)	7 (12.1)	1 (1.7)	58 (100)
NBUVB	21 (45.7)	15 (32.6)	1 (2.2)	1 (2.2)	7 (15.2)	1 (2.2)	46 (100)
PUVA	3 (50.0)	0 (0.0)	1 (16.7)	0 (0.0)	0 (0.0)	2 (33.3)	6 (100)
Radiation therapy	2 (28.6)	1 (14.3)	1 (14.3)	1 (14.3)	1 (14.3)	1 (14.3)	7 (100)
Chemotherapy	0 (0.0)	2 (22.2)	1 (11.1)	0 (0.0)	4 (44.4)	2 (22.2)	9 (100)
Topical chlormethine	2 (33.3)	2 (33.3)	0 (0.0)	0 (0.0)	1 (16.7)	1 (16.7)	6 (100)
Duration of treatment (months), mean (range)	34 (3-168)	26 (1-168)	14 (4-31)	72 (72-72)	34 (3-74)	24 (5-48)	31 (1-168)
Duration of follow-up (months), mean (range)	34 (1-108)	32 (1-72)	19 (0-48)	72 (72-72)	49 (3-144)	32 (12-48)	35 (0-144)
***Outcomes***							
Complete remission	7 (63.6)	3 (27.3)	1 (9.1)	0 (0.0)	0 (0.0)	0 (0.0)	11 (100)
Partial response	6 (31.6)	8 (42.1)	0 (0.0)	1 (5.3)	2 (10.5)	2 (10.5)	19 (100)
Stable disease	16 (53.3)	9 (30.0)	2 (6.7)	0 (0.0)	3 (10.0)	0 (0.0)	30 (100)
Progressive disease	3 (30.0)	3 (30.0)	0 (0.0)	0 (0.0)	3 (30.0)	1 (10.0)	10 (100)
Relapse	2 (66.7)	0 (0.0)	0 (0.0)	0 (0.0)	1 (33.3)	0 (0.0)	3 (100)
***Mortality***							
Deceased	0 (0.0)	0 (0.0)	1 (33.3)	0 (0.0)	0 (0.0)	2 (66.7)	3 (100)

NBUVB: narrow-band ultraviolet B, PUVA: psoralen plus ultraviolet-A

**Figure 2:**
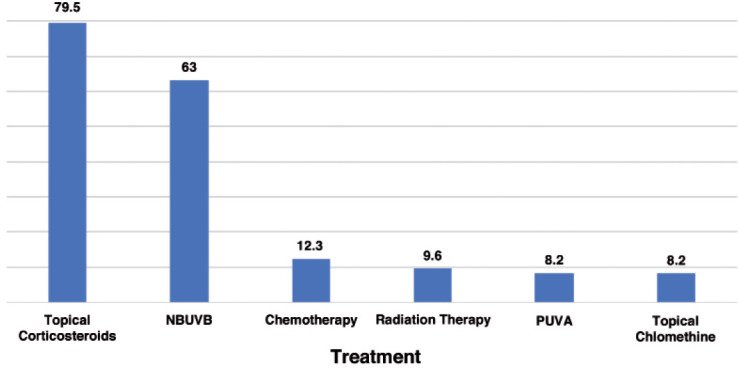
- Kaplan–Meier survival curve of early MF (stages IA, IB, and IIA) and advanced MF (stages IIB, III, IVA, and IVB).

The difference in the survival distribution for the 2 TNMB stages of MF, early and advanced, was assessed using the Kaplan–Meier estimator for survival (Figure 2). In early-stage MF, the disease-specific survival (DSS) rate was 98.3%, while in advanced-stage MF, the DSS rate was 84.6% (*p*=0.032). The overall survival of all MF patients was 95.9%.

## Discussion

Mycosis fungoides is a pronounced entity, yet reports on its clinical characteristics vary among different populations. Thus far, few reports on the profile of MF in Arab patients have been published. Studies on the histology and prognosis of MF are especially lacking in Saudi Arabia. In our center, the mean age at diagnosis was 44 years, which is older than previously reported in local studies by Alghamdi et al^10^ (33.5 years) and Binamer et al^11^ (41 years) but younger than reports from the USA (57 years) and the Netherlands (61 years).^8,12^ This supports the suggestion of an earlier-onset of MF in this population. Twenty-three percent of our patients are aged 30 years or younger, including 4 children. This percentage is similar to corresponding percentages from published international reports but is less than half the reported percentage locally by Alghamdi et al^10^ (48.8%). The male-to-female ratio was 1:1.3, which is inconsistent with the local and international figures that typically show a male predominance. The reason for the slight female predominance in our study is unclear.

Mycosis fungoides can present in various morphologies, posing a diagnostic challenge. Therefore, many patients are labeled with the wrong diagnosis before confirming MF. In our analysis, 20% of the patients were initially misdiagnosed with atopic dermatitis. This might be explained by the shared clinical presentation of pruritic, erythematous, scaly plaques between the 2 entities. The clinically equivocal cases coupled with the initial misdiagnoses may prolong MF diagnosis. We found that our dermatologists reached the MF diagnosis within a mean of 33 months (2.75 years). This duration is less than previously reported in the United States (U.S.), which showed a mean duration of 4.2 years from cutaneous symptoms to MF diagnosis.^12^

Consistent with the published literature, classic MF (patches or plaques) was the most prevalent presentation. Hypopigmented MF was observed in 20.5% of our patients. This percentage was higher than previously reported by Binamer et al^11^ (15.2%) but far less than that reported by Alghamdi et al^10^ (42%). This could be due to the dissimilarity in age between our patients and those of Alghamdi et al,^10^ as the hypopigmented variant is more common among younger patients. However, all local percentages of hypopigmented MF were higher than international figures, which supports the belief that it is more common among patients with darker skin complexions. A total of 82% percent of our patients had early-stage MF (such as: IA, IB, and IIA), which is comparable to the work of Alghamdi et al^10^ (83.3%) but higher than findings in the U.S. (66%)and Korea (76.4%).^12,13^

The immunohistochemical profile in our study matched the pattern reported by Moon et al^13^ in 97 Korean patients (the majority of tumor cells were CD4 positive). Interestingly, our analysis showed that patients with CD4+/CD8+ with CD8+ predominance generally had better clinical courses and outcomes compared to patients with CD4+ predominance. However, the use of cell surface antigens to predict the prognosis remains a point of debate. According to a number of studies, the MF cell phenotype is not a factor that influences the prognosis, while others suggest that a predominance of CD8 positivity predicts a slower progression of the disease.^14-16^ Martinez et al^17^ concluded in their study that predominantly CD8+ MF patients carry a favorable prognosis with a negligible risk for disease progression. In a case series carried out by Nikolaou et al,^18^ all patients with CD8-positive MF had an indolent course, suggesting that CD8+ cytotoxic immunophenotype could constitute a marker of mild biological behavior. Despite the lack of consensus, our results support the claim that CD8+ predominant MF confers a better clinical course and outcomes.

Large cell transformation is the histopathologic transformation of more than 25% of the neoplastic lymphocytes into clonally identical, larger lymphocytes (example, 4 times the previous size).^19^ The overall prevalence of LCT among MF patients is reported within the range of 8-55%.^20^ In our study, LCT was present in 4 patients (5.5%), all of whom were CD30 positive. This was lower than previously reported in 187 American patients by Talpur et al^21^ (9.8%). The diagnosis of LCT is historically considered a marker of poor prognosis; however, recent studies have disputed the affirmation that LCT forecasts MF prognosis. In our patients, the presence of LCT was not linked to poor clinical course and outcomes. This is similar to an analysis by Agar et al,^22^ which found that LCT and tumor distribution may influence the disease course but not survival outcomes. In contrast, other studies revealed that patients with LCT exhibit an aggressive clinical course and poor survival.^23,24^ It is evident from the results of our study and prior reports that not all patients with transformed MF experience adverse clinical outcomes and poor survival. Therefore, other factors, such as clinical stage, may be an explanation for disease aggression in transformed MF rather than LCT alone.^25^

The most frequently prescribed treatment across all stages of MF in our study was topical corticosteroids (79.5%). This is incompatible with a study by Doorn et al,^8^ who reported different therapies depending on stage in Dutch patients. They showed that psoralen plus ultraviolet-A (PUVA) was more commonly prescribed in early-stage MF (55%), while radiotherapy was frequently used in late-stage MF (49%). However, this can be explained by the unavailability of PUVA in our center in the last several years due to the increased use of NBUVB. Of note, some common treatment modalities, such as oral bexarotene, are unavailable in our center.

Mycosis fungoides patients may develop extracutaneous involvement that can potentially be fatal. The prevalence of death related to MF among our patients was 4.1%. This was lower than previous reports, including the Dutch study in which death reached 15% among 309 patients.^8^ Among our patients, a substantial difference in the DSS rate between the 2 MF stages was noted. The DSS rate in the early-stage MF was 98.3%, while in advanced-stage MF, the DSS rate dropped to 84.6%. The survival rate of the Saudi population presented in this cohort remained higher than observed in Europe but slightly lower than that seen in China, where it was 98.6% for early-stage MF and 88.9% for advanced-stage MF.^8,9^ Nonetheless, the favorable DSS among our patients was likely influenced by the high percentage (78%) of early-stage MF patients compared to the advanced stage.

### Study limitation

Overall, this study was limited by its single-center approach, relatively small number of subjects, and lack of prognostic factors analysis that may play a role in the overall survival. We suggest a multicenter nationwide study on MF among the Saudi population.

In conclude, our results show that among Saudi patients, MF has an earlier onset and a higher prevalence in females. Hypopigmented MF is more prevalent in our ethnic group. Immunohistochemical staining of CD4+/CD8+ with CD8 predominance may elucidate a favorable disease course. Finally, despite advances in dermatology, the time between symptoms onset and diagnosis, although shorter than previous reports, still falls short of expectations when it comes to early cancer detection. A thorough understanding of the clinical variants of MF could aid in the prompt diagnosis and initiation of early therapy, leading to better overall outcomes.
